# Pooled genome wide association detects association upstream of *FCRL3* with Graves’ disease

**DOI:** 10.1186/s12864-016-3276-z

**Published:** 2016-11-18

**Authors:** Jwu Jin Khong, Kathryn P. Burdon, Yi Lu, Kate Laurie, Lefta Leonardos, Paul N. Baird, Srujana Sahebjada, John P. Walsh, Adam Gajdatsy, Peter R. Ebeling, Peter Shane Hamblin, Rosemary Wong, Simon P. Forehan, Spiros Fourlanos, Anthony P. Roberts, Matthew Doogue, Dinesh Selva, Grant W. Montgomery, Stuart Macgregor, Jamie E. Craig

**Affiliations:** 1 Melbourne Medical School Western Campus, Department of Medicine, University of Melbourne, Sunshine Hospital, 176 Furlong Road, St Albans, VIC 3021 Australia; 2 Orbital, Plastics and Lacrimal Unit, The Royal Victorian Eye and Ear Hospital, Heidelberg, VIC Australia; 3 Department of Ophthalmology and Department of Surgery, University of Melbourne, Austin Health, Heidelberg, VIC Australia; 4 Menzies Institute for Medical Research, University of Tasmania, Hobart, TAS Australia; 5 Statistical Genetics, Queensland Institute of Medical Research (QIMR) Berghofer Medical Research Institute, Herston, QLD Australia; 6 Department of Ophthalmology, Flinders University of South Australia, Bedford Park, South Australia Australia; 7 Department of Surgery, Centre for Eye Research Australia and Ophthalmology, University of Melbourne, East Melbourne, VIC Australia; 8 Department of Endocrinology and Diabetes, Sir Charles Gairdner Hospital, Nedlands, WA Australia; 9 School of Medicine and Pharmacology, The University of Western Australia, Crawley, WA Australia; 10 Centre for Ophthalmology and Visual Sciences, University of Western Australia, Western Australia, Australia; 11 Department of Medicine, School of Clinical Sciences, Monash University, Clayton, VIC Australia; 12 Department of Endocrinology and Diabetes, Western Health, St Albans, VIC Australia; 13 Department of Diabetes and Endocrinology, Royal Melbourne Hospital, Parkville, VIC Australia; 14 Department of Endocrinology, The Royal Adelaide Hospital, Adelaide, South Australia Australia; 15 Department of Medicine, University of Otago, Christchurch, New Zealand; 16 South Australian Institute of Ophthalmology, University of Adelaide, South Australia, Australia; 17 Molecular Epidemiology, Queensland Institute of Medical Research (QIMR) Berghofer Medical Research Institute, Herston, QLD Australia

**Keywords:** Graves’ disease, Genome-wide association study, Pooled blood

## Abstract

**Background:**

Graves’ disease is an autoimmune thyroid disease of complex inheritance. Multiple genetic susceptibility loci are thought to be involved in Graves’ disease and it is therefore likely that these can be identified by genome wide association studies. This study aimed to determine if a genome wide association study, using a pooling methodology, could detect genomic loci associated with Graves’ disease.

**Results:**

Nineteen of the top ranking single nucleotide polymorphisms including HLA-DQA1 and C6orf10, were clustered within the Major Histo-compatibility Complex region on chromosome 6p21, with rs1613056 reaching genome wide significance (*p* = 5 × 10^−8^). Technical validation of top ranking non-Major Histo-compatablity complex single nucleotide polymorphisms with individual genotyping in the discovery cohort revealed four single nucleotide polymorphisms with *p* ≤ 10^−4^. Rs17676303 on chromosome 1q23.1, located upstream of *FCRL3,* showed evidence of association with Graves’ disease across the discovery, replication and combined cohorts. A second single nucleotide polymorphism rs9644119 downstream of *DPYSL2* showed some evidence of association supported by finding in the replication cohort that warrants further study.

**Conclusions:**

Pooled genome wide association study identified a genetic variant upstream of *FCRL3* as a susceptibility locus for Graves’ disease in addition to those identified in the Major Histo-compatibility Complex. A second locus downstream of *DPYSL2* is potentially a novel genetic variant in Graves’ disease that requires further confirmation.

**Electronic supplementary material:**

The online version of this article (doi:10.1186/s12864-016-3276-z) contains supplementary material, which is available to authorized users.

## Background

Graves’ disease (GD) is an autoimmune thyroid disease which typically presents as thyrotoxicosis and goitre; other clinical findings include Graves’ orbitopathy, dermopathy and acropachy. The incidence rate of GD is 15 per 100, 000 persons per year with an overall mean prevalence of 1152 per 100, 000 population in the United States [1, 2]. Women are disproportionately more affected with the female to male ratio reported at 7.3:1 [1]. The disease also occurs in children at one tenth the prevalence rate of all ages [1].

GD is a complex disease with a strong familial predisposition; [3–7] the triggers are poorly understood but various environmental factors were linked to onset of GD such as stress, smoking and *Yersinia* infection in genetically susceptible individuals [8, 9]. GD shows a strong familial predisposition. The concordance rate for GD in monozygotic twins is significantly higher than dizygotic twins and model-fitting analysis predicted that genetic factors account for 79 % of disease heritability in twins [10]. With a concordance rate of 22–35 % for monozygotic twins, environmental and modifier factors are likely to play a key role [10, 11]. Clustering of GD with other autoimmune conditions including type I diabetes mellitus, myasthenia gravis, polymyositis, lupus erythematosus and Addison’s disease suggest genes regulating the immune system shared by these diseases might also contribute towards the genetic susceptibility for GD [3].

The genetic susceptibility of GD can be broadly categorized into genes involved in immune regulation, and thyroid specific genes. Genes confirmed to be associated with GD include the following: multiple loci within the Human leukocyte antigen (HLA) I and II region, as well as non-HLA loci *CTLA4, PTPN22, CD40, IL2A, FCRL3* and *TSHR* [12–26]. The contributions from each of these genetic variants are modest. From candidate gene studies, HLA class II alleles DRB1*03(DR3), DQA1*0501, DQB1*02 were significantly associated with GD with odd ratios of 2 to 3 for *DR3* [5, 15, 27, 28]. *CTLA4* A49G, and CT60 3′UTR polymorphisms were more frequent in GD with an odds ratio of 1.4 to 2, [12, 13, 29–31] and association with *PTPN22* polymorphism has an odds ratio of 1.5 to 1.9 [14, 16, 17].

Genome wide association studies (GWAS) have replicated earlier candidate gene study findings and identified additional new genetic loci. The earliest GWAS studies on autoimmune thyroid disease showed association of GD with multiple loci within the HLA class I and II regions, and also in *TSHR* and *FCRL3* [26, 32]. More recently two new risk loci for GD, the *RNASET2-FGFR1OP-CCR6* region at 6q27 and an intergenic region at 4p14 were reported, along with confirmation of positive association of GD with SNPs in HLA*-DPB1, TSHR, CLTA4* and *FCRL3* [33]. Using a custom array, association of SNPs near *PTPN22, CTLA4, TSHR, RNASET2-FGFR1OP-CCR6* loci with GD were replicated, and seven novel susceptibility loci, *MMEL1*, *LPP*, *BACH2*, *PRICKLE1*, *ITGAM*, rs1534422 at 2p25.1 and rs4409785 at 11q21 were identified but the results require confirmation in replication studies [24].

A pooled GWAS strategy can effectively identify gene variants in multiple diseases. Using relatively small numbers of cases and controls, pooled GWAS has previously been used to identify the known susceptibility loci *CFH* and *ARMS2/HTRA1* in age related macular degeneration and *LOXL1* in pseudoexfoliation syndrome [34]. A pooled DNA study has also identified a new susceptibility locus, *HGF*, in keratoconus (a progressive corneal degeneration) and additional novel candidate genes related to platelet reactivity in diabetic patients [34–36]. The study design must be carefully considered to include multiple pools to minimise pooling errors and an appropriate array platform to extract maximal available information from the pooled DNA [37, 38].

The aim of this study was to determine if GWAS using DNA pooling methodology could replicate genomic loci associated with GD and discover new genetic loci.

## Methods

### Definition of Graves’ disease

GD cases were participants with biochemically confirmed hyperthyroidism on thyroid function tests; and had either the presence of Graves’ orbitopathy, or positive thyrotropin receptor (TSHR) antibodies or uniform increase in uptake on technetium-99 m pertechnetate thyroid scan.

### Discovery cohort

The Australian Thyroid-associated Orbitopathy Research (ATOR) discovery cohort consisting of 412 GD cases with and without Graves’ orbitopathy of European descent was recruited from endocrinology and ophthalmology clinics in the hospitals and private practices from Victoria and South Australia.

A total of 498 controls were recruited from two Australian populations. These comprised 198 adult patients with keratoconus, a non-autoimmune eye disease unrelated to GD, who were recruited from eye clinics in Melbourne, and 300 healthy controls aged over 50 years recruited from retirement villages and the general community in South Australia. The control cohort was not actively screened for GD, but none of the controls reported having this condition.

### Replication cohort

An independent group of 539 GD cases and 1230 controls were recruited to form a replication cohort. The GD cases were new cases recruited into the ATOR cohort (*n* = 373) from Melbourne, and endocrine and ophthalmology clinics from Western Australia (*n* = 166). The controls were research participants of European descent with eye diseases without recognizable association with GD or other autoimmune diseases (angle closure glaucoma or suspect, pigment dispersion syndrome, steroid responsive ocular hypertension, myopia and keratoconus) recruited in a collaborative effort from South Australia and Victoria to study the genetics of these eye diseases (age ranging from 9 to 92 years).

### DNA pooling for GWAS study

Two methods of DNA pooling were used. For freshly collected samples, whole blood was pooled directly (pool 1) and for archived DNA samples, equimolar DNA pools were created (pools 2 and 3). Venous blood or DNA aliquots from all participants were thawed for 3 h before construction of 3 pool comparisons. Pool number 1 contained 154 GD cases and 198 controls, pool number 2 had 218 GD cases and 213 controls, and pool number 3 had 40 GD cases and 87 controls, giving a total of 412 cases and 498 controls from the discovery cohort. For pool 1, 100 μl of whole blood from each sample was mixed with protease and lysis buffer, vortexed and incubated, then samples were combined in one tube for whole blood pool construction, 1 tube each for cases and controls. DNA was extracted from the whole blood pool by using QIA-amp DNA blood maxi kit (Qiagen, Valencia, CA, USA) according to the manufacturer’s protocol. For pool 2 and 3 samples, whole blood was lysed and DNA was extracted from each sample. Equimolar DNA pools were constructed in duplicate by step-wise dilutions of genomic DNA using QIA-amp DNA blood maxi kit (Qiagen, Valencia, CA, USA) to 75 ng/μl. We used Fluoroskan Ascent microplate fluorometer (ThermoFisher Scientific, Waltham, MA, USA) coupled with Picogreen reagents (Invitrogen, Carlsbad, CA) for DNA quantitation. Comparisons were made to a standard curve generated from serial dilutions of Lambda DNA (Invitrogen, Carlsbad, CA). A total of 451 ng of DNA from each sample was added to the equimolar DNA pool.

### Pooling error

Previous studies showed that the majority of pooling errors arise from errors on the arrays rather than pool construction errors, [39] hence in this pooling design, multiple Illumina HumanOmni5-Quad arrays were used for each pool to reduce array errors. The pooling standard deviation is very small for the Illumina bead arrays, pooling standard deviation for HumanHap300 was estimated between 0.007 and 0.011, [38] and variance for the 1M array was 6.8 × 10^−5^ [34]. This encompasses both assays and pool construction errors. The Illumina HumanOmni5-Quad beadchip array supersedes its predecessor arrays; the HumanHap300 and 1M array, by virtue of its comprehensive coverage of the human genome but is similar in the way of the bead array design.

### Pooled genotyping and analysis

Genome-wide genotyping was conducted at Queensland Institute of Medical Research (QIMR) Berghofer Medical Research Institute, using HumanOmni5-Quad beadchip microarray (Illumina, San Diego, CA, USA) which contained approximately 4.3 million markers. Three case and control pools, each replicated 2 to 4 times to minimise measurement error, were generated for comparisons.

The output of the raw beadscores from Illumina Beadstation were subjected to a series of data cleaning and quality control measures, as described previously for array-based DNA pooling studies [38, 40, 41]. SNPs with minor allele frequency (MAF) greater than 1 % from the reference panel of EUR samples in the 1000 Genomes Project were retained for subsequent analysis (i.e. 2.5 million SNPs). We excluded SNPs with more than 10 % negative beadscores, and SNPs with the sum of mean red and green scores smaller than 1200 across each array. A correction factor (*corr*) was calculated to rescale the green beadscore to calibrate the mean value of the pooling allele frequency (PAF) at 0.5 over all SNPs on each strand of the array. The PAF was computed based on the raw red intensities and the corrected green intensities as PAF = red/(red + green/*corr*) [40]. We further filtered non-autosomal SNPs and any SNPs with a significant variance difference between cases and controls.

A final set of autosomal SNPs meeting the following criteria: (1) contain sufficient number of probes (i.e., over ½ of expected number of probes for SNPs with MAF 1–5 %, or over 1/3 of expected number of probes for SNPs with MAF >5 %; (2) display no larger than 0.3 differences between PAF and reference allele frequency from EUR samples in the 1000 Genomes Project were taken forward for association testing. After data cleaning and quality control, 2.2M SNPs were left for association testing. A linear model was used to test for allelic association in the three case-control pools from GD discovery cohort. The test statistic corrected for pooling error was used to rank SNPs; with *p* values derived from chi-squared distribution with 1 ° of freedom to assess the significance of allele frequency difference between cases and controls. We then conducted a meta-analysis weighted by inverse variance to combine the results of the three case-control pool comparisons. The top-ranking SNPs in the meta-analysis were counter checked for proxies (i.e., linkage disequilibrium *r* ^2^ >0.5 with the index SNPs in the EUR populations). If proxy SNPs were available, they should also show evidence of association to be considered valid. A genome-wide significance threshold was set at 5 × 10^−8^ to account for multiple testing but SNPs with *p* < 1 × 10^−6^ were also included for further evaluation.

### Individual genotyping and analysis

Top-ranked non-major histo-compatability complex (MHC) SNPs with *p*-value ≤1 × 10^−6^ were selected for technical validation genotyping in individual DNA samples that made up the pool as well as for genotyping in the replication cohorts. The top ranking SNPs were individually genotyped in the discovery cohort in 412 GD cases and 480 normal controls. A subset of 18 controls had insufficient residual samples for individual genotyping, hence the discrepancy in the final pooling and validation sample sizes.

Thirteen SNPS were genotyped on iPLEX Gold assay (Sequenom, San Diego, CA, USA) by using Sequenom MassARRAY® Analyzer in two batches. The ATOR discovery cohort and Western Australia replication cohort genotyping was performed at the Australian Genome Research Facility (Brisbane, Australia) and the ATOR replication cohort at GeneWorks Pty Ltd (Adelaide, Australia). The allele association analysis was performed using chi-square test (−−assoc) in PLINK, [42] independently for technical validation in the ATOR pooled discovery cohort, the replication cohorts and the overall in the discovery and replication cohorts. The odds ratio and 95 % confidence interval for allelic association was further adjusted for covariates age and sex using logistic regression analysis (−−logistic −−covar). SNPs with *p* ≤ 1 × 10^−4^ were considered validated in validation genotyping. For replication genotyping, *p* <0.05 was considered significant.

## Results

The GD cases were predominantly female and younger when compared with controls (Table 1), hence subsequent individual genotyping analysis was adjusted for age and sex.

**Table 1:** Demographics of Graves’ disease cases and controls in the discovery and replication cohorts

	N		Mean age (SD)			Sex		
	Case	Control	Case	Control	*p* value	Graves’ case	Controls	*p* value
Discovery cohort	412	498	45.3 (14.5)	56.3 (24.4)	<0.001	Male 71 (17.23 %)	Male 192 (41.03 %)	<0.001
						Female 341 (82.77 %)	Female 276 (58.97 %)	
Replication cohort	539	1230	44.5 (14.8)	48.1 (17.4)	<0.001	Male 109 (20.30 %)	Male 596 (49.30 %)	<0.001
						Female 428 (79.70 %)	Female 613 (50.70 %)	

In the discovery GWAS, 19 of the top 30 ranking SNPs clustered within the MHC region on chromosome 6p21 including rs9272937 in HLA-DQA1 (p 10^−7^), two SNPs within C6orf10 (p 10^−7^), and rs1613056 reaching genome wide significance (*p* = 5 × 10^−8^) (Additional file 1: Table S1). One SNP, rs9676286 on chromosome 19, reached genome-wide significance in the pooled GWAS (*p* = 1.08 × 10^−8^) for association with GD. However rs9676286 was not as strongly supported when we conducted technical validation genotyping of the individual DNA samples from the pools (*p* = 5.54 × 10^−3^).

An additional 12 top ranked non-MHC SNPs suggestive of association (p ~ 10^−6^) were also selected for validation. Of these, four SNPs showed evidence of association with *p* < 1 × 10^−4^ when we conducted technical validation genotyping in the individual DNA samples. They are rs1469893 on chr 15, rs17676303 on chr 1, rs78542322 on chr 6 and rs62469516 on chr 7 (Table 2). Amongst these four, rs1767303 was most strongly associated with GD with a *P* value at 1.14 × 10^−8^ (OR = 2.05, 95 % CI 1.57–2.67). When correlating odds ratio of effect allele frequencies in pooled GWAS with individual validation genotyping, the results showed strong correlation (*r* = 0.89, *p* <0.001) (Additional file 2: Figure S1).

**Table 2:** Top ranking SNPs identified from pooled genome wide association study and validation genotyping in the discovery cohort

			GWAS pooled genotyping (Cases = 412, Controls = 498)			Validation individual genotyping (Cases = 412, Controls = 480)			
SNP name	Location chr and bp	Gene	A1	*P* value	OR_A1	F_A	F_U	*P* value*	OR_A1* (95 % CI)
Rs9676286	Chr19q13.43 58126481	ZNF134 intron	A	1.08 × 10^−8^	2.08	0.047	0.023	5.54 × 10^−3^	2.28 (1.27, 4.09)
rs9644119	Chr8p21.2 26517817	DPYSL2 2124 bp downstream	T	9.10 × 10-^8^	1.78	0.177	0.128	0.033	1.36 (1.03, 1.79)
rs11722643	Chr4p16.1 10127484	Intergenic WDR1 upstream, ZNF518B downstream	T	1.03 × 10^−7^	1.88	0.101	0.054	1.38 × 10^−3^	1.91 (1.28, 2.83)
Rs686806	Chr 11q22.3 107228533	CWF19L2 intron	T	2.36 × 10^−7^	1.70	0.242	0.168	9.6 × 10^−4^	1.53 (1.19, 1.97)
rs62469516	Chr7q21.13 88958624	ZNF804B intron	A	2.68 × 10^−7^	0.51	0.043	0.088	5.73 × 10^−5^	0.41 (0.26, 0.63)
rs73452600	Chr 7q34 142828104	PIP 1070 bp upstream	A	3.49 × 10^−7^	1.90	0.099	0.061	3.37 × 10^−3^	1.80 (1.22, 2.67)
Rs1662312	Chr 18p11.31 3220450	MYOM1 upstream	A	5.72 × 10^−7^	0.51	0.040	0.080	5.33 × 10^−4^	0.45 (0.29, 0.71)
rs1469893	Chr 15q14 34988664	GJD2 56015 bp Downstream	A	6.58 × 10^−7^	0.58	0.05	0.104	6.86 × 10^−5^	0.44 (0.29, 0.66)
Rs17676303	Chr 1q23.1 157679691	FCRL3 9044 bp upstream	T	8.05 × 10^−7^	1.68	0.254	0.150	1.14 × 10^−8^	2.05 (1.57, 2.67)
rs28578508	Chr18q12.1 32316007	DTNA intron	A	8.16 × 10^−7^	0.56	0.062	0.099	2.80 × 10^−3^	0.56 (0.39, 0.82)
rs78542322	Chr 6q14.1 77291670	Intergenic IMPG1 upstream HTR1B downstream	T	9.82 × 10^−7^	0.52	0.071	0.141	3.27 × 10^−5^	0.47 (0.33, 0.67)
Rs3818779	Chr 10q26.11 121140671	GRK5 intron	A	1.51 × ^−6^	1.94	0.059	0.029	2.08 × 10^−3^	2.30 (1.35, 3.9)
rs2141440	Chr 11q14.3 90898463	Intergenic MIR4490 and FAT3	A	4.48 × 10^−6^	0.58	0.078	0.097	0.34	0.84 (0.59, 1.20)

F_U is individual genotyping minor allele frequency A1 in normal control, F_A is the allele frequency in Graves’ disease

OR is odd ratio for having minor allele in Graves’ cases compared to normal controls

*P values and odds ratios in validation genotyping were adjusted for age and sex

Genotyping in the replication cohort showed positive association of GD with rs17676303, located upstream of *FCRL3* on chromosome 1q23.1 (OR = 1.22, 95 % CI 1.01–1.46, *p* = 0.04). The minor T allele was more frequent in GD cases than in normal controls (OR = 1.42, 95 % CI 1.22–1.64, *p* = 3.41 × 10^−6^) in the combined discovery and replication cohorts, adjusted for sex and age. Rs62469516, rs1469893 and rs78542322 minor alleles were not associated with GD in the replication cohort, or the combined cohorts (Table 3).

**Table 3:** Genotyping results for top ranking single nucleotide polymorphisms in replication cohorts, and combined analysis of discovery and replication cohorts using individual genotyping

			Replication cohorts (Cases = 539, Controls = 1230)					Combined discovery and replication cohorts (Cases = 951, Controls = 1710)			
SNP name	Location chr and bp	Gene	A1	F_A	F_U	*P* value	OR_A1 (95 % CI)	F_A	F_U	*P* value*	OR_A1*
Rs9676286	Chr19q13.43 58126481	ZNF134 intron	A	0.045	0.039	0.41	1.17 (0.81–1.71)	0.046	0.034	0.04	1.38 (1.02, 1.87)
rs9644119	Chr8p21.2 26517817	DPYSL2 2124 bp downstream	T	0.219	0.174	9.34 × 10^−3^	1.28 (1.06–1.54)	0.200	0.161	4.02 × 10^−3^	1.25 (1.07, 1.45)
rs11722643	Chr4p16.1 10127484	Intergenic WDR1 upstream, ZNF518B downstream	T	0.096	0.095	0.94	0.99 (0.76–1.29)	0.098	0.083	0.14	1.17 (0.95, 1.44)
Rs686806	Chr 11q22.3 107228533	CWF19L2 intron	T	0.198	0.210	0.95	0.99 (0.82, 1.20)	0.217	0.197	0.04	1.63 (1.004, 1.35)
rs62469516	Chr7q21.13 88958624	ZNF804B intron	A	0.053	0.053	0.64	1.09 (0.77–1.53)	0.048	0.063	0.06	0.77 (0.59, 1.01)
rs73452600	Chr 7q34 142828104	PIP 1070 bp upstream	A	0.090	0.084	0.71	1.05 (0.81, 1.36)	0.094	0.077	0.07	1.22 (0.98–1.50)
Rs1662312	Chr 18p11.31 3220450	MYOM1 upstream	A	0.070	0.070	0.77	0.93 (0.59, 1.50)	0.049	0.073	1.72 × 10^−3^	0.61 (0.44, 0.83)
rs1469893	Chr 15q14 34988664	GJD2 56015 bp Downstream	A	0.074	0.081	0.72	0.95 (0.71, 1.27)	0.064	0.088	7.29 × 10^−3^	0.73 (0.57, 0.92)
Rs17676303	Chr 1q23.1 157679691	FCRL3 9044 bp upstream	T	0.233	0.201	0.04	1.22 (1.01–1.46)	0.242	0.186	3.41 × 10^−6^	1.42 (1.22, 1.64)
rs28578508	Chr18q12.1 32316007	DTNA intron	A	0.069	0.083	0.13	0.80 (0.60, 1.07)	0.066	0.087	3.21 × 10^−3^	0.71 (0.56, 0.89)
rs78542322	Chr 6q14.1 77291670	Intergenic IMPG1 upstream HTR1B downstream	T	0.094	0.096	0.89	0.98 (0.76, 1.27)	0.084	0.109	9.45 × 10^−3^	0.76 (0.62, 0.93)
Rs3818779	Chr 10q26.11 121140671	GRK5 intron	A	0.055	0.049	0.58	1.10 (0.78, 1.55)	0.057	0.043	0.06	1.31 (0.99, 1.72)
rs2141440	Chr 11q14.3 90898463	Intergenic MIR4490 and FAT3	A	0.094	0.086	0.53	1.09 (0.84, 1.41)	0.087	0.089	0.88	0.98 (0.80, 1.21)

*P values and odds ratios are adjusted for age and sex

We further examined the relationship between SNP rs17676303 and other annotated SNPs in *FCRL3* gene or its promoter region, namely rs7528684, rs3761969 and rs11264798, which have previously been identified as positively associated with GD, rheumatoid arthritis and systemic lupus erythematosus [25, 33, 43]. All three SNPs previously associated with GD showed high linkage disequilibrium with rs17676303 in the Caucasian HapMap population (Table 4). The result suggests that these SNPs reported with various auto-immune diseases may be tagging the same functional genetic variants around the *FCRL3* gene locus.

**Table 4:** Haploview linkage disequilibrium between known susceptibility loci in Graves’ disease and rs17676303 identified in pooled GWAS for Graves’ disease in Hapmap CEU population data

Locus 1	Locus 2	D’	Log of the Odds (LOD)	R^2^	CI low	CI High
rs11264798	rs17676303	1.0	4.44	0.142	0.64	1.0
rs3761959	rs17676303	1.0	5.83	0.221	0.71	1.0
rs7528684	rs17676303	1.0	5.73	0.224	0.7	1.0

A second SNP, rs9644119, located downstream of *DPYSL2* on chromosome 8p21 is highly ranked for its association with GD in pooled GWAS with a *P* value approaching genome wide significance (*p* = 9.10 × 10^−8^). The association was not validated in the discovery cohort and for the combined cohort the association with GD is less strong than the discovery pooled GWAS would suggest (*p* = 4.02 × 10^−3^). In the replication cohort, the minor T allele conferred increased odds for GD compared to controls (OR = 1.33, 95 % CI 1.11–1.59, *p* = 1.95 × 10^−3^); after adjusting for sex and age, the result remained significant. (OR = 1.28, 95 % CI 1.06–1.54, *p* = 9.34 × 10^−3^). The results suggested this allele might be a genetic locus of interest for GD, but will need further confirmation in future studies.

## Discussion

This study investigated the genetic associations of GD using a pooled genome wide association study (GWAS) in 910 samples. Despite the relatively small number of samples for discovery GWAS, the pooled strategy in combination with increased sample size in a second stage of individual genotyping in both the validation and replication cohorts, readily detected evidence of association of a genetic locus upstream of *FCRL3* on chromosome 1q23. The association of *FCRL3* with GD has previously been identified in large scale GWAS studies [25, 26, 33]. The pooled GWAS also detected association of GD with multiple SNPs within the MHC I and MHC II regions on chromosome 6p21. Susceptibility loci within both these regions which contain HLA genes involved in antigen presentation, are well established for GD by association studies, candidate gene analysis and GWAS [5, 25–27, 44]. Rs9272937 in the un-translated region of *HLA-DQA1* within the MHC class II region showed evidence of association with GD (*p* = 4.43 × 10^−7^) in our study. The positive association of *HLA-DQA1* with GD has been replicated in multiple studies [15, 44, 45]. Two SNPs within the intron of the *C6orf10* gene suggesting association with GD (p 10^−7^), were also independently validated as a locus of susceptibility in GD in a previous GWAS study [26].

These replicative findings in GD prove the robustness of the two-stage study design for pooled GWAS. The pooled GWAS study also found evidence of association for a gene locus marked by rs9644119, downstream of *DPYSL2* on chromosome 8, but the significance is less certain. The risk attributions from individual genetic variants for GD are known to be small with odds ratios <1.5, [23] therefore large sample sizes in the thousands will be needed to increase power for detecting significant association, and may in the future prove or disprove the significance for the rs9644119 locus.

For the two compelling signals we have detected in the vicinity of *FCRL3* and *DPYSL2*, genetic variants in *FCRL3* were associated with a modest increase in odds for GD at 1.2 to 1.35 [25, 33, 46]. Genetic variation in the promoter region of *FCRL3* i.e. *FCRL3* -169T>C allele is reportedly associated with other autoimmune diseases such as rheumatoid arthritis and systemic lupus erythematosus, [43, 47, 48] suggesting that the *FCRL3* locus may influence the susceptibility to multiple autoimmune diseases in a similar manner to the immune regulating *CTLA4, PTPN22* and MHC genes [24, 25, 32]. To pinpoint the exact locus associated with *FCRL3*, the entire *FCRL* region on chromosome 1q21 was previously investigated using imputation, and the strongest signals associated with GD were located in a cluster of SNPs including rs7528684 and rs3761959 [49]. SNP rs7528684 in the promoter region of *FCRL3* was also previously reported as positively associated with rheumatoid arthritis, systemic lupus erythematosus and GD [43]. Rs3761959 tagging rs7522061 and rs7528684 in the *FCRL3* gene showed association with GD in the Wellcome Trust Case Control Consortium GWAS study (*p* = 9.4 × 10^−3^), and the association was further confirmed by The China Consortium for the Genetics of Autoimmune Thyroid disease, reaching genome wide significance (*p* = 2.22 × 10^−8^) [25, 33]. In addition, rs11264798 in *FCRL3* was also associated with GD (*p* = 1.6 × 10^−5^) [25]. We found high linkage disequilibrium of rs17676303, the SNP associated with GD in our study, with rs7528684, rs3761959 and rs11264798, that strongly suggest these SNPs were tagging the same functional variants in the vicinity of the *FCRL3* gene.

Functional analysis suggests FCRL3 has immune regulating roles. FCRL3 is an Fc receptor like molecule that shares a genomic location (chr 1q21-23) and gene structure as a receptor for the Fc portion of immunoglobulin with other members of the immunoglobulin superfamily [50]. Unlike the Fc receptor, the FCRL molecule has not been shown biochemically to bind immunoglobulin [50]. FCRL3 molecules are preferentially expressed by B cells [51, 52]. FCRL3 possesses both tyrosine based activating and inhibitory motifs in its cytoplasmic tail, indicating these may be used to transmit immune-modulatory signals via tyrosine phorphorylation in regulating B cell differentiation and response [50]. The strongest evidence linking *FCRL3* polymorphisms with autoimmunity is derived from linkage disequilibrium mapping of the chr1q21-23 region in rheumatoid arthritis, which identified 4 SNPs (rs7528684, rs11264799, rs945635 and rs3761959) in a Japanese population [43]. Of the 4 SNPs positively associated with rheumatoid arthritis, *FCRL3* -169T>C (marked by rs7528684 in the promoter region of *FCRL3*) substitution substantially increased promoter activity in *FCRL3* and enhanced binding affinity to the consensus NF-κβ binding motif, where NF-κβ widely regulates genes involved in immune response [43]. In addition, *FCRL3* transcripts in peripheral B cells and genomic DNA are higher in heterozygous individuals with genotype -169C/T +358C/G, and increased FCRL3 expression in synovial tissue and peripheral CD19+ B cell population suggest that higher expression of FCRL3 is potentially pathogenic [43]. Further correlation of B cell abnormalities with FCRL3 expression is afforded by significant positive correlation of rheumatoid factor autoantibody titre in individuals with rheumatoid arthritis with the number of susceptible −169 C alleles [43]. However not all studies showed consistent association with polymorphisms in *FCRL3*, specifically no discernible association with *FCRL3* were noted in some other rheumatoid arthritis populations including Caucasians in North American and European countries [53–55]. When the susceptibility effect of *FCRL3* was stratified by NF-κβ1 genotypes, association of *FCRL3* polymorphisms with rheumatoid arthritis was observed in patients heterozygous for the NF-κβ1 promoter in a Spanish population [54].

The second gene of interest, *DPYSL2*, produces collapsin response mediator protein 2 (CRMP2). It was first described in the central nervous system as an effector cytosolic protein for semaphorin mediated axonal growth cones navigation during neural development and plasticity [56]. Dysregulation of CRMP2 phosphorylation and expression, have been associated with neuropsychiatric diseases including Alzheimer’s disease and schizophrenia [56]. CRMP2 is also highly expressed in activated T lymphocytes from peripheral blood bearing CD69 and HLA-DR markers. CRMP2 is distributed in the trailing uropod in polarized T cells binding to cytoskeletal structures particularly to vimentin [57]. The observations of enhanced spontaneous and chemokine-directed T cell transmigration by induced expression of CRMP2 in Jurkat T cells, and high expression of CRMP2 in migrating T cells suggest CRMP2 may regulate T cell motility and migration [57]. The mechanism underlying CRMP2 induced T cell migration seems to involve phosphorylation of CRMP2 Tyr-479 residue, acting as transducer for chemokine stromal cell-derived factor-1α (CXCL12) signaling [58]. The discovery of CRMP2 cellular function in T cells defines its potential role in the pathogenesis of immune mediated inflammation, specifically neuroinflammation [59].

## Conclusions

In conclusion, the pooled GWAS is an effective methodology for detecting common genetic variants in GD. The study confirmed *FCRL3* region as a susceptibility locus for GD in addition to MHC. A second locus downstream of *DPYSL2* is potentially a novel genetic variant in GD that requires further confirmation.

## Additional files

Additional file 1: Table S1. Top 30 single nucleotide polymorphism associated with Graves’ disease compared with controls in genome wide association study. This table denoted the top ranking SNPs and highlighted SNPs in major histocompatibility complex region and non-HLA SNPs that were chosen for subsequent validation. (DOCX 141 kb)
Additional file 2: Figure S1. Scatterplot for odds ratio of 13 effect allele frequencies comparing pooled GWAS Graves’ disease with individual genotyping in the discovery cohort. This figure showed the correlation between odds ratio derived from pooled GWAS genotyping and individual genotyping in the ATOR discovery cohort; the correlation is high *r* = 0.89. (TIF 9765 kb)

## Abbreviations

ATOR: Australian thyroid-associated orbitopathy research
GD: Graves’ disease
GWAS: Genome wide association study
HLA: Human leukocyte antigen
MAF: Minor allele frequency
MHC: Major histo-compatibility complex
PAF: Pooling allele frequency
SNP: Single nucleotide polymorphism
TSHR: Thyrotropin receptor
