# Supplementary material for: Pooled genome wide association detects association upstream of FCRL3 with Graves’ disease
Source: BMC Genomics. 2016 Nov 18;17:939. doi: 10.1186/s12864-016-3276-z (PMC5116198; doi:10.1186/s12864-016-3276-z)
Supplement: Additional file 1: Table S1. — Top 30 single nucleotide polymorphism associated with Graves’ disease compared with controls in genome wide association study. This table denoted the top ranking SNPs and highlighted SNPs in major histocompatibility complex region and non-HLA SNPs that were chosen for subsequent validation. (DOCX 141 kb) [file 12864_2016_3276_MOESM1_ESM.docx]

Supplementary table 1. Top 30 single nucleotide polymorphism associated with Graves’ disease compared with controls in genome wide association study.

| SNP name | Chr | BP position | Effect allele | Effect allele frequency | OR | P-value |
| --- | --- | --- | --- | --- | --- | --- |
| rs9676286* | 19 | 58126481 | A | 0.1441 | 2.08 | 1.08x10^-8^ |
| rs1613056^†^ | 6 | 32668946 | T | 0.2313 | 1.87 | 5.04x10^-8^ |
| rs9644119* | 8 | 26517817 | T | 0.297 | 1.78 | 9.10x10^-8^ |
| rs11722643* | 4 | 10127484 | T | 0.1938 | 1.88 | 1.03x10^-7^ |
| Rs2395149^†^ | 6 | 32325562 | A | 0.2517 | 1.76 | 1.14x10^-7^ |
| rs2098230 | 4 | 10242924 | A | 0.9001 | 0.47 | 1.21x10^-7^ |
| Rs2647044^†^ | 6 | 32667910 | A | 0.2737 | 1.74 | 1.41x10^-7^ |
| rs674313^†^ | 6 | 32578082 | T | 0.3748 | 1.67 | 1.49x10^-7^ |
| Rs3132090^†^ | 6 | 31430752 | A | 0.2627 | 1.78 | 1.85x10^-7^ |
| rs3131379^†^ | 6 | 31721033 | A | 0.2381 | 1.76 | 2.25x10^-7^ |
| rs686806* | 11 | 107228533 | T | 0.3005 | 1.70 | 2.36x10^-7^ |
| rs62469516* | 7 | 88958624 | A | 0.2349 | 0.51 | 2.68x10^-7^ |
| Rs9272190^†^ | 6 | 32601760 | A | 0.7595 | 0.57 | 2.90x10^-7^ |
| Rs2395228^†^ | 6 | 32623223 | A | 0.8464 | 0.53 | 2.92x10^-7^ |
| rs73452600* | 7 | 142828104 | A | 0.1889 | 1.90 | 3.49x10^-7^ |
| rs3132445^†^ | 6 | 31712196 | A | 0.2398 | 1.73 | 3.58x10^-7^ |
| rs9272937^†^ | 6 | 32611040 | A | 0.3969 | 1.64 | 4.43x10^-7^ |
| Rs9266001^†^ | 6 | 31316695 | A | 0.2301 | 1.74 | 5.20x10^-7^ |
| Rs3117109^†^ | 6 | 32340871 | T | 0.253 | 1.72 | 5.49x10^-7^ |
| rs1662312* | 18 | 3220450 | A | 0.2089 | 0.51 | 5.72x10^-7^ |
| Rs1265757^†^ | 6 | 32302382 | T | 0.231 | 1.72 | 6.37x10^-7^ |
| Rs2517597^†^ | 6 | 30081189 | A | 0.2414 | 1.72 | 6.51x10^-7^ |
| rs1469893* | 15 | 34988664 | A | 0.3369 | 0.58 | 6.58x10^-7^ |
| Rs3099844^†^ | 6 | 31448976 | A | 0.2668 | 1.68 | 6.96x10^-7^ |
| Rs313595^†^ | 6 | 32405192 | T | 0.4687 | 0.61 | 7.44x10^-7^ |
| Rs2844559^†^ | 6 | 31340075 | T | 0.2834 | 1.68 | 7.79x10^-7^ |
| rs17676303* | 1 | 157679691 | T | 0.29 | 1.68 | 8.05x10^-7^ |
| rs28578508* | 18 | 32316007 | A | 0.273 | 0.56 | 8.16x10^-7^ |
| Rs642093^†^ | 6 | 32582075 | A | 0.3439 | 1.64 | 9.66x10^-7^ |
| Rs9272275^†^ | 6 | 32603603 | T | 0.3366 | 1.64 | 9.74x10^-7^ |
| rs78542322* | 6 | 77291670 | T | 0.2644 | 0.52 | 9.82x10^-7^ |
| rs3818779* | 10 | 121140671 | A | 0.1235 | 1.94 | 1.51x10^-6^ |
| rs2141440* | 11 | 90898463 | A | 0.2653 | 0.58 | 4.48x10^-6^ |

*denotes top ranking non-HLA SNPs that were chosen for validation, rs 2098230 was not typed due to failure to design the probe on Sequenom multiplex.

^†^ denotes SNPs in major histocompatibility complex region.
