# Supplementary figures and images for: Pooled genome wide association detects association upstream of FCRL3 with Graves’ disease
Source: BMC Genomics. 2016 Nov 18;17:939. doi: 10.1186/s12864-016-3276-z (PMC5116198; doi:10.1186/s12864-016-3276-z)

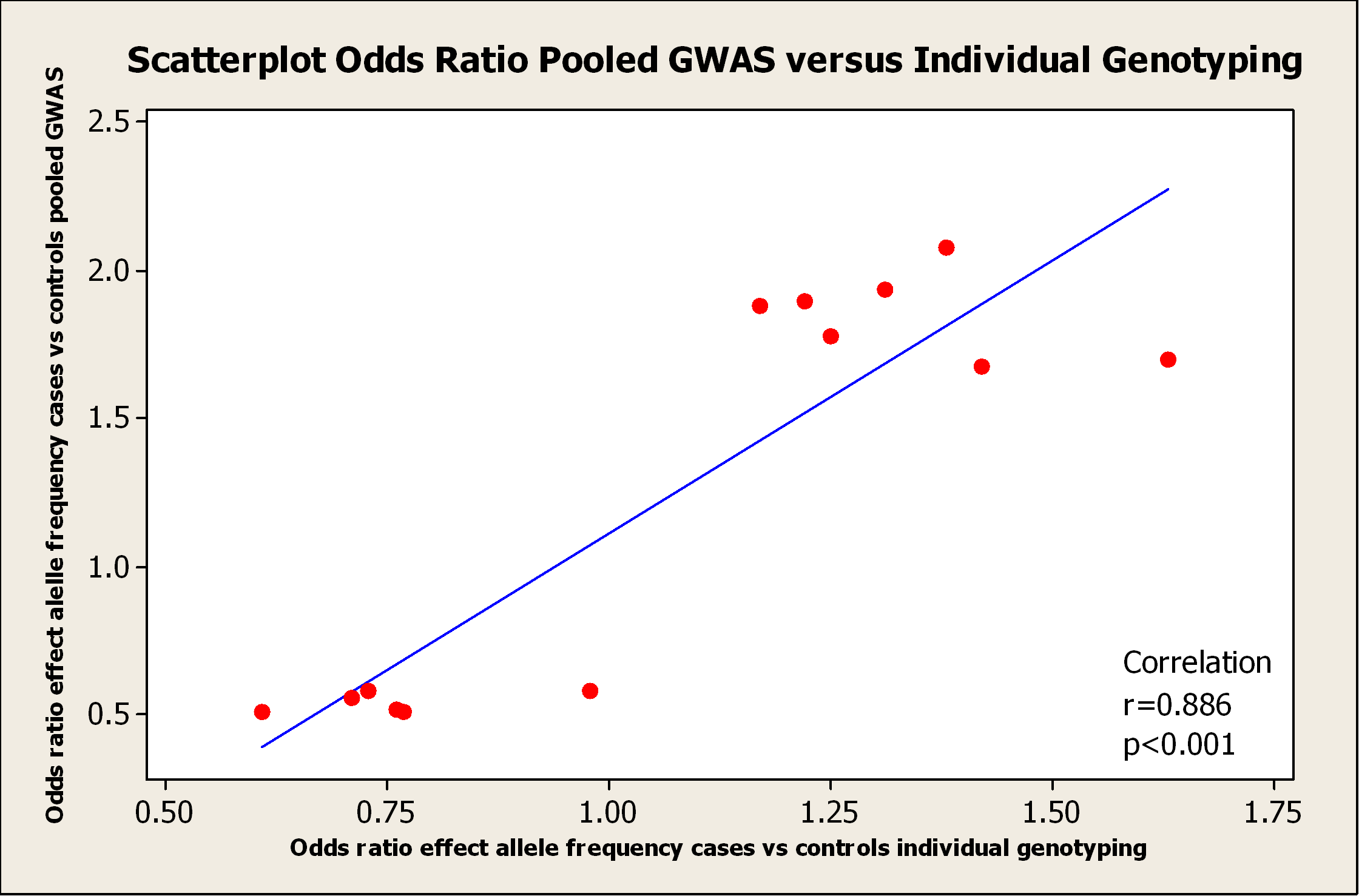

Supplement: Additional file 2: Figure S1. — Scatterplot for odds ratio of 13 effect allele frequencies comparing pooled GWAS Graves’ disease with individual genotyping in the discovery cohort. This figure showed the correlation between odds ratio derived from pooled GWAS genotyping and individual genotyping in the ATOR discovery cohort; the correlation is high r = 0.89. (TIF 9765 kb) [file 12864_2016_3276_MOESM2_ESM.tif]
